# Genome Sequence of Two Novel Species of Torque Teno Minivirus from the Human Oral Cavity

**DOI:** 10.1128/genomeA.00868-14

**Published:** 2014-09-25

**Authors:** Marcos Parras-Moltó, Patricia Suárez-Rodríguez, Asier Eguia, José Manuel Aguirre-Urizar, Alberto López-Bueno

**Affiliations:** a Centro de Biología Molecular Severo Ochoa (Consejo Superior de Investigaciones Científicas-Universidad Autónoma de Madrid), Madrid, Spain; b Departamento de Estomatología II, UFI 11/25 Universidad del País Vasco/EHU, Leioa, Spain

## Abstract

*Anelloviridae* is a family of circular, single-stranded DNA viruses highly prevalent among humans. We report the genome sequence of two torque teno miniviruses found in human oral mucosa samples. Genome organization, phylogenetic analysis, and pairwise comparisons reveal that they belong to novel species within the *Betatorquevirus* genus.

## GENOME ANNOUNCEMENT

*Anelloviridae* is a highly divergent family of viruses with circular single-stranded DNA genomes. Three genera of anelloviruses are able to infect humans: torque teno virus (TTV; *Alphatorquevirus*), torque teno minivirus (TTMV; *Betatorquevirus*), and torque teno minivirus (TTMDV; *Gammatorquevirus*), with genome sizes of 3.8 to 3.9 kb, 2.8 to 2.9 kb, and 3.2 kb, respectively (1). Anellovirus genome is composed of a noncoding G+C-rich region and three open reading frames (ORFs) encoding up to six different proteins through alternative splicing (2). They are disease-orphan viruses, showing high prevalence and mixed infections in humans (3). Total virus burden in blood increases upon immunosuppression (4), so that anellovirus load has been proposed as a subrogated marker for immunocompetence status (5, 6). The oral cavity might play an important role in anellovirus transmission since these viruses have been frequently detected in respiratory tract fluids (7, 8) and saliva (9). Indeed, anelloviruses can reach higher titers in saliva than in serum (10), and the same genotype can be found in the serum and saliva of the same person (9).

We have carried out a large metagenomic survey (Illumina; MiSeq; 2×300 bp) of DNA viruses from the oral vestibule mucosa of nine healthy adults and oral ulcers of seven individuals affected by recurrent aphthous stomatitis. After assembling quality-filtered (PrinSeq; minimum quality, ≥25 phred score) sequences with CLC Genomic Workbench-6 (http://www.clcbio.com), we found 17 contigs of >2 kbp that showed BLASTx similarities to anelloviruses, including two circular genomes with best BLAST similarities to viruses from the *Betatorquevirus* genus. The first one, tentatively designated TTMV_ALH8, was assembled with coverage ×35 from a healthy oral mucosa sample and spans 2,855 nucleotides with 38.77% G+C content. The second one, TTMV_ALA22, was attained with ×18 coverage from an oral ulcer sample and spans 2,914 nucleotides with 38.37% G+C contents. These genome sizes and % G+C contents are in the range of those reported for available *Betatorquevirus* (1). Additionally, TTMV_ALH8 and TTMV_ALA22 exhibit typical TTMV genome organization characterized by a region with high G+C content, a large ORF1 (656 and 674 amino acids [aa], respectively), a small ORF2 (89 and 110 aa, respectively), and a small ORF3 overlapping the 3′ end of ORF1. The lack of initial ATG in the ORF3 of TTMV-ALH8 has been reported for other TTMVs (11). A comprehensive phylogenetic analysis based on available ORF1 aa sequences (R; package *ade4*, unrooted maximum likelihood tree) clustered TTMV-ALH8 together with TTMV_LY1 (accession no. JX134044), TTMV_D11 (KF764701), and TTMV_D50 (KF764702), while TTMV_ALA22 grouped with TTMV_9 (AB038631) and TTMV_8 (AF291073).

The current criterion demarcating species within the *Anelloviridae* family consist of at least 35% nucleotide divergence in pairwise comparisons of the ORF1 sequence (http://www.ictvonline.org/). Alignments obtained with Clustal-Omega (http://www.clustal.org) classified 15 out of the 17 ORF1 sequences found in contigs within previously established species, 12 of them into the *Alphatorquevirus* genus. On the contrary, nucleotide sequences of TTMV_ALH8 and TTMV_ALA22 ORF1 share 59.11% and 60.68% identity to the closest related anelloviruses (TTMV_LY1 and TTMV_9, respectively), meeting the criteria to be proposed as novel species of the *Betatorquevirus* genus.

### Nucleotide sequence accession numbers.

The complete genome sequences of torque teno minivirus ALH8 and torque teno minivirus ALA22 have been deposited in GenBank under accession numbers KM259874 and KM259873, respectively.

## References

[1] Biagini P 2009 Classification of TTV and related viruses (anelloviruses). Curr. Top. Microbiol. Immunol. 331:21–33. 10.1007/978-3-540-70972-5_2 19230555

[2] Qiu J Kakkola L Cheng F Ye C Söderlund-Venermo M Hedman K Pintel DJ 2005 Human circovirus TT virus genotype 6 expresses six proteins following transfection of a full-length clone. J. Virol. 79:6505–6510. 10.1128/JVI.79.10.6505-6510.2005 15858033 PMC1091685

[3] Hino S Miyata H 2007 Torque teno virus (TTV): current status. Rev. Med. Virol. 17:45–57. 10.1002/rmv.524 17146841

[4] Li L Deng X Linsuwanon P Bangsberg D Bwana MB Hunt P Martin JN Deeks SG Delwart E 2013 AIDS alters the commensal plasma virome. J. Virol. 87:10912–10915. 10.1128/JVI.01839-13 23903845 PMC3807392

[5] De Vlaminck I Khush KK Strehl C Kohli B Luikart H Neff NF Okamoto J Snyder TM Cornfield DN Nicolls MR Weill D Bernstein D Valantine HA Quake SR 2013 Temporal response of the human virome to immunosuppression and antiviral therapy. Cell 155:1178–1187. 10.1016/j.cell.2013.10.034 24267896 PMC4098717

[6] Focosi D Maggi F Albani M Macera L Ricci V Gragnani S Di Beo S Ghimenti M Antonelli G Bendinelli M Pistello M Ceccherini-Nelli L Petrini M 2010 Torquetenovirus viremia kinetics after autologous stem cell transplantation are predictable and may serve as a surrogate marker of functional immune reconstitution. J. Clin. Virol. 47:189–192. 10.1016/j.jcv.2009.11.027 20034850

[7] Galmès J Li Y Rajoharison A Ren L Dollet S Richard N Vernet G Javouhey E Wang J Telles JN Paranhos-Baccalà G 2013 Potential implication of new torque teno mini viruses in parapneumonic empyema in children. Eur. Respir. J. 42:470–479. 10.1183/09031936.00107212 23060626 PMC3729974

[8] Maggi F Pifferi M Fornai C Andreoli E Tempestini E Vatteroni M Presciuttini S Marchi S Pietrobelli A Boner A Pistello M Bendinelli M 2003 TT virus in the nasal secretions of children with acute respiratory diseases: relations to viremia and disease severity. J. Virol. 77:2418–2425. 10.1128/JVI.77.4.2418-2425.2003 12551979 PMC141071

[9] Ross RS Viazov S Runde V Schaefer UW Roggendorf M 1999 Detection of TT virus DNA in specimens other than blood. J. Clin. Virol. 13:181–184. 10.1016/S1386-6532(99)00015-3 10443794

[10] Deng X Terunuma H Handema R Sakamoto M Kitamura T Ito M Akahane Y 2000 Higher prevalence and viral load of TT virus in saliva than in the corresponding serum: another possible transmission route and replication site of TT virus. J. Med. Virol. 62:531–537. 10.1002/1096-9071(200012)62:4<531::AID-JMV20>3.3.CO;2-3 11074484

[11] Biagini P Gallian P Attoui H Touinssi M Cantaloube J de Micco P de Lamballerie X 2001 Genetic analysis of full-length genomes and subgenomic sequences of TT virus-like mini virus human isolates. J. Gen. Virol. 82:379–383 11161277 10.1099/0022-1317-82-2-379

